# Equity of Elderly Care Facility Allocation in a Multi-Ethnic City under the Aging Background

**DOI:** 10.3390/ijerph20043291

**Published:** 2023-02-13

**Authors:** Haolin He, Yujia Chen, Yaxin Liu, Yang Gu, Ying Gu

**Affiliations:** 1 Faculty of Business Administration, Osaka University of Economics, Osaka 533-8533, Japan; 2 School of Architecture, Tianjin University, Tianjin 300072, China; 3 Faculty of Food Science and Engineering, Kunming University of Science and Technology, Kunming 650500, China

**Keywords:** aging, multi-ethnic city, elderly care facilities, spatial distribution, equity

## Abstract

Societal concerns in ethnic minority areas are global issues. Paying close attention to the equitable allocation of social resources in an aging population is crucial to preserving the cultural diversity and social stability of multi-ethnic countries. This study took a multi-ethnic city—Kunming (KM), China—as an example. The population aging and the comprehensive service level of elderly care institutions at the township (subdistrict) scale were evaluated to discuss the equity of elderly care facility allocation. This study revealed that the overall convenience of elderly care institutions was low. The coupling coordination between the degree of aging and the service level of elderly care institutions in the majority of areas in KM showed poor adaptation. There is spatial differentiation in population aging and an imbalanced distribution of elderly care facilities and relevant service facilities among ethnic minority communities and other areas in KM. We also attempted to provide optimization recommendations for existing problems. This study, on the degree of population aging, the service level of elderly care institutions, and the degree of coupling coordination between them at the township (subdistrict) scale, offers a theoretical foundation for planning elderly care facilities in multi-ethnic cities.

## 1. Introduction

Aging is a trend of population development and a long-term challenge worldwide, especially for populous countries, such as China. The seventh National Census of China in 2021 indicated that the number of individuals aged 60 and above reached 260 million, representing 18.70% of the total population [1]. With the continuously increasing aging population, the demand for elderly services has increased, bringing new challenges to China’s elderly care system. The degree of coupling coordination between the supply of elderly care resources and the degree of population aging is connected to the allocation efficiency of social-related resources and whether or not the elderly have equitable access to elderly care services.

Relevant research has demonstrated a correlation between the economic development of nations or regions and the equity of the distribution of elderly care facilities [2]. China is a country with a sizable population and 56 different ethnic groups. As most of the population belongs to the Han ethnic group, China’s other 55 ethnic groups are considered ethnic minorities. Unlike in the developed areas in China, most multi-ethnic areas show significant supply-and-demand mismatches in elderly care resources and difficulty in providing elderly care services because of their underdeveloped economic conditions, unique geographic position, cultural background, and autonomous system. For example, according to the Ethnic and Religious Affairs Commission of Yunnan Province [3], in 2021, the per capita disposable income of permanent rural residents in ethnic autonomous areas of Yunnan Province was CNY 14,166, much less than the per capita disposable income of Yunnan residents (CNY 25,666) in 2021. Studying population aging, assessing the spatial distribution of elderly care institutions and infrastructure, and analyzing the service quality of elderly care institutions could help in developing a valid strategy for allocating elderly care resources rationally and dealing with population aging effectively.

Researchers from many countries have studied the spatial planning of elderly care institutions and have proposed optimization strategies. Japanese researchers analyzed the spatial distribution of the local elderly population in detail and investigated care facilities for the elderly [4] and the living environment of elderly individuals that live alone [5] and then proposed aging-friendly optimization strategies in terms of daily living facilities for the elderly, street-related facilities, public transportation facilities, hospital beds, and disaster response [6,7,8,9]. Western researchers also proposed the healthcare desert theory [10], which illustrates the correlation between the level of medical services and the distribution of the aging population and explores the impact of accessibility to public facilities [11] and medical facilities [12] based on the regional population. Chinese researchers explored the application and effect of GIS in solving the aging problem [13]. The spatial distribution of elderly care resources in different provinces and cities [14,15,16,17,18,19] was analyzed to explore reasonable planning solutions for elderly facilities. Moreover, some researchers evaluated the accessibility of living service facilities [20,21,22], parks and green spaces [23], and medical facilities [24] in different provinces and cities and explored the coordination between supply and demand and the rationality of the allocation of living service facilities for the elderly population. Studies on the elderly populations of ethnic minorities in China have also been carried out. Chinese scholars explored the relationship between social support and life satisfaction among the elderly of ethnic minorities [25] and evaluated their health-related quality of life (Yi) in Yunnan [26]. However, the discussion on the equality of the allocation of elderly facilities in multi-ethnic cities is limited.

This study evaluated the degree of population aging, the level of basic services, and the convenience of living in elder care institutions in KM at the township (subdistrict) scale. The equity of the distribution of elderly care facilities in ethnic minority communities and other areas in KM was discussed, and optimization suggestions were also proposed.

## 2. Materials and Methods

### 2.1. Study Area

**Figure 1:**
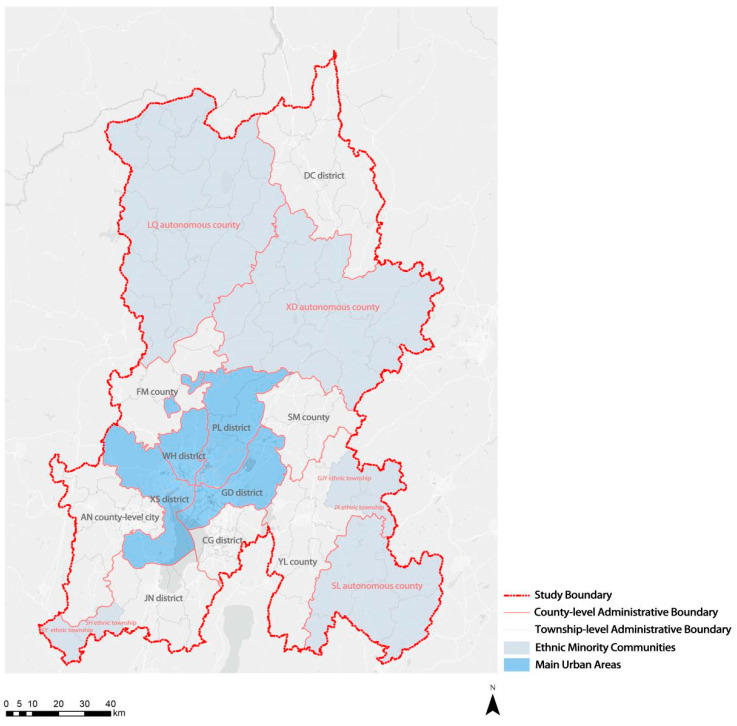
Study area (different districts/counties and townships/subdistricts are marked).

The study area in this study covered seven districts (Wuhua (WH) District, Panlong (PL) District, Guandu (GD) District, Xishan (XS) District, Dongchuan (DC) District, Chenggong (CG) District, and Jinning (JN) District), three counties (Fumin (FM) County, Yiliang (YL) County, and Songming (SM) County), three autonomous counties (Shilin (SL) Autonomous County, Luquan (LQ) Autonomous County, and Xundian (XD) Autonomous County), and one county-level city (Anning (AN)) in KM. The abbreviations of all administrative districts and townships mentioned in our article are listed in Table A1. As the provincial capital of Yunnan Province, KM is an important central city in western China, with an area of approximately 21,000 square kilometers, including 3 autonomous ethnic minority counties, 4 ethnic minority townships, and 2196 villages with populations of a mixed ethnic origin. The population of ethnic minorities is 1.3 million, accounting for 18.9% of the city’s population, and ethnic minority communities comprise 57% of the city’s land area. Three autonomous counties and four ethnic townships constitute the main ethnic minority communities. Twenty-four ethnic minority groups live in LQ Autonomous County, accounting for 29.95% of the total population. Twenty-six ethnic minority groups live in SL Autonomous County, and the ethnic minority population accounts for 33.52% of the total population. Twenty-seven ethnic minority groups are concentrated in XD Autonomous County, accounting for 22.46% of the total population. As the city with the largest number of ethnic autonomous areas and the largest number of hereditary ethnic components among the provincial capitals in China (Figure 1), population aging in ethnic minority communities is a focus issue for KM, alongside aging in the general population.

### 2.2. Data

#### 2.2.1. Data Sources

This study was based on data from the seventh population census conducted by the KM Bureau of Statistics. The evaluation indices associated with population aging, including the proportion of different age groups and the elderly dependency ratio, were calculated. Web crawler technology was used to crawl the data of elderly care institutions and point of interest (POI) facilities and information on the walking paths of elderly care facilities to surrounding POI services from the websites of “linkolder”, “Elderly Care Information Network”, and “AMAP”. Compared with the previous spatial accessibility evaluation standard, which is based on spatial distance, this study used the AMAP open platform and time accessibility as the evaluation standard, which is closer to the reality of residents’ daily routines. AMAP is currently the largest digital map platform in China and has the most advanced transportation algorithm. It has world-leading accuracy in calculations. The specific method utilized is as follows: use the path planning tool of AMAP, select the “walking” mode of travel, take each elderly care institution as the starting point, take each POI service facility as the end point, and input the corresponding coordinates into the program to obtain the time needed to reach each POI from each elderly care facility, and then carry out statistical analysis. After data cleaning and calculation, ArcGIS (10.8) was used to evaluate the comprehensive service level of elderly care institutions in KM by analyzing the fundamental service level and living convenience.

#### 2.2.2. Web Crawler Technology

**Figure 2:**
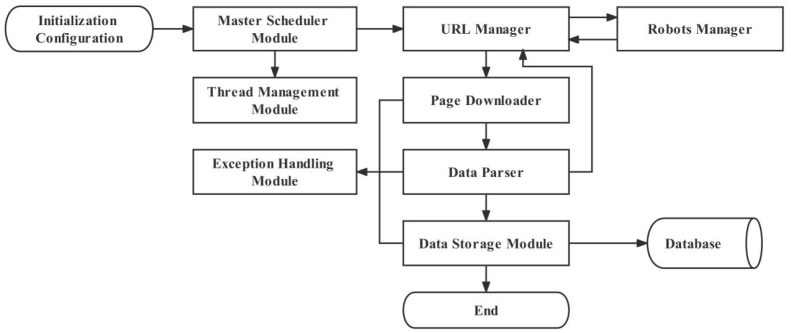
Flow chart of web information crawling.

The web crawler system [27] consists of eight functional parts: system scheduling, URL link management, web page download, web page parsing, data storage, robot management, thread management, and risk management. Figure 2 depicts the flow chart of web information crawling in this study.

#### 2.2.3. Entropy Method

Information entropy can be used to calculate the objective weights of each index to avoid bias caused by subjective evaluation. This study used the entropy method to calculate the weights of the evaluation indices of the degree of aging and the service level of elderly care institutions. The specific calculation formula [28] is as follows:

(1) $$S_{j}=\sum_{j}^{m}W_{j}P_{\mathrm{ij}}\times 100,$$

where S_j_ is the composite score of the jth indicator, W_j_ refers to the weight of indication, and P_ij_ represents the weight of indication in the ith area.

#### 2.2.4. Space Adaptation Analysis

The coupling coordination model involves a coupling degree—C, coordination degree—D, and coordination index—T, which indicate the level of coupling coordination between two or more systems. This study investigated the adaptation degree between the degree of population aging and the service levels of elderly care institutions. The obtained data were first normalized to eliminate the difference in quantitative units between the two systems, thus ensuring the trustworthiness of the analysis results. The calculation formula [29] is as follows:

(2) $${Χ}_{i}^{*}=\{\begin{array}{c} \frac{X_{i}-X_{\min}}{X_{\max}-X_{\min}}\\ \frac{X_{\max}-X_{i}}{X_{\max}-X_{\min}} \end{array}$$

where ${Χ}_{i}^{*}$ and X_i_ represent the values before and after data normalization, respectively, and X_min_ and X_max_ correspond to the minimum and maximum values in the sample data, respectively.

C represents the system’s coupling degree, which can be calculated using Formulas (3) and (4).

(3) $$U_{i}=\sum_{j=1}^{n}\partial_{\mathrm{ij}}X_{\mathrm{ij}}\;,\sum_{j=1}^{n}=1,\;i=1,2,$$

where U_i_ is the comprehensive evaluation index, U1 corresponds to the comprehensive score for the degree of population aging, U2 is the comprehensive score for the service level of elderly care institutions, and $\partial_{\mathrm{ij}}$ corresponds to the weight of each index.

(4) $$C=\frac{2\sqrt{\left(U_{1}U_{2}\right)}}{U_{1}+U_{2},}$$

When C = 0, no correlation is found between the involved systems. The correlation between the systems increases as C increases. When C = 1, the involved systems show the highest degree of coupling.

The coupling coordination degree model was introduced to further evaluate the coordination between the two systems (i.e., the degree of population aging and the service level of elderly care institutions). The formulas of the coupling coordination degree model are as follows:

(5) $$D=\sqrt{C\times T}$$

(6) $$T={\mathrm{aU}}_{a}\times {\mathrm{bU}}_{S},\;a+b=1$$

where D represents the coupling coordination degree, C is the coupling degree, and T corresponds to the comprehensive evaluation index of the degree of population aging and the service level of elderly care institutions, which is determined based on $U_{a}$ and $U_{S}$. a and b in Formula (6) refer to the evolution indices of $U_{a}$ and $U_{S}$, respectively.

In order to visually describe the adaptation between the service level of elderly care institutions and the degree of population aging, the level of the coupling coordination degree was rated on a scale of 0–10 (Table A2). All statistical analyses in this study were carried out using SPSS statistics 23.0.

## 3. Results and Discussion

### 3.1. Degree of Population Aging

There are nearly 1.22 million people over 60-years-old in KM, accounting for 14.40% of the total population. The number of people over 65-years-old is almost 890,000, accounting for 10.45% of the total population, which indicates that KM has entered a mild aging stage. The current status of population aging in the city of KM was further investigated. The visualized data suggest a significant difference in the population aging level between ethnic minority communities and other areas in KM. The distribution regularities are summarized as follows.

#### 3.1.1. Low Population Aging but High Density of the Elderly Population in Central Urban Areas

**Figure 3:**
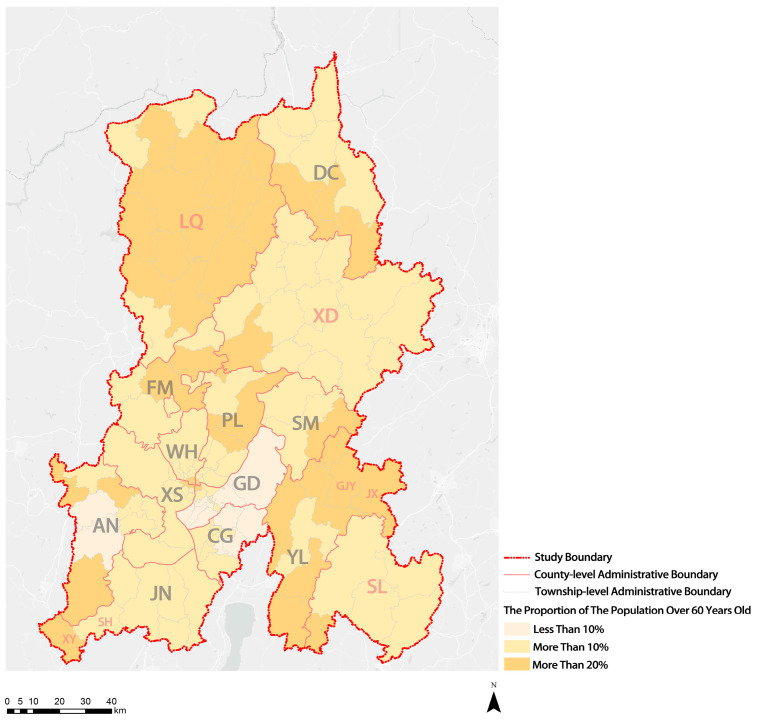
Visualization of distribution results of percentage of the elderly population in KM.

As illustrated in Figure 3, compared with other administrative regions at the same level, the central urban areas of KM showed lower population aging. The number of individuals aged 60 or older in the majority of townships (subdistricts) in the three central urban areas (WH District, XS District, and PL District) of KM exceeded 10% of the total population, entering the stage of light aging. The number of individuals aged 60 or older in the majority of townships (subdistricts) in the GD District (one of the central urban areas) did not reach 10% of the total population, which suggests that it has not yet entered the aging stage. In a small part of the First Ring Road, the over-60-year-old population accounted for more than 20% of the total population, demonstrating that it was entering a moderate aging stage.

**Figure 4:**
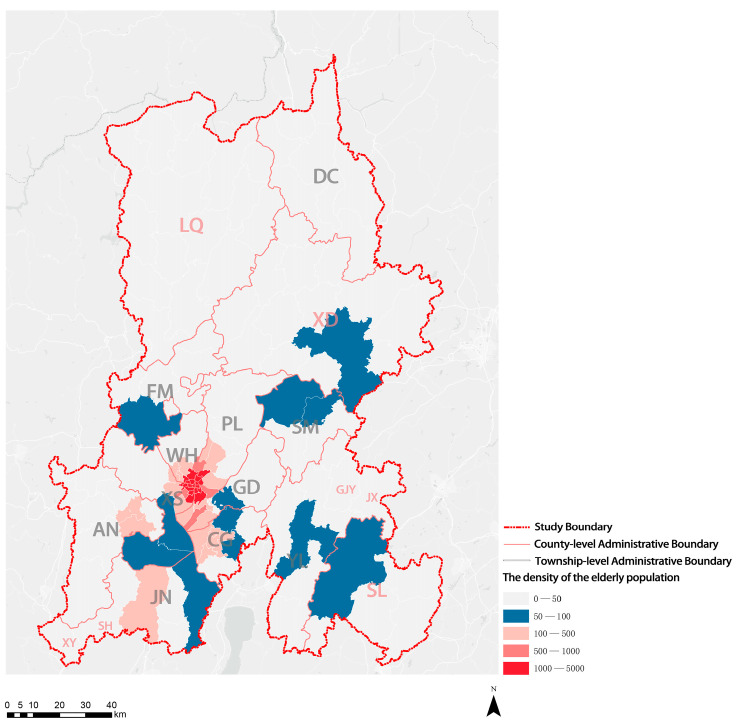
Visualization of distribution results of population density of the elderly population in KM.

The density of the elderly population is an important index used to evaluate the spatial difference in population aging. Given the shortage of land in the central area of KM, the urban service function is highly concentrated, and the number of elderly individuals per unit area increases accordingly. Therefore, the urban service function and the density of the elderly population were high in the center and low in the surrounding areas (Figure 4).

#### 3.1.2. High Degree of Population Aging and High Dependency Burden for the Working Population in Ethnic Minority Communities

Overall, the rate of population aging was higher in ethnic minority communities than in other areas in KM. LQ Autonomous County had the highest degree of aging of the three autonomous counties. Except for three townships (subdistricts), the number of individuals aged 60 or older in townships (subdistricts) of LQ Autonomous County exceeded 20% of the total population, showing moderate aging. In addition, one township in XD Autonomous County and SL Autonomous County and four ethnic townships in KM had entered mild aging.

**Figure 5:**
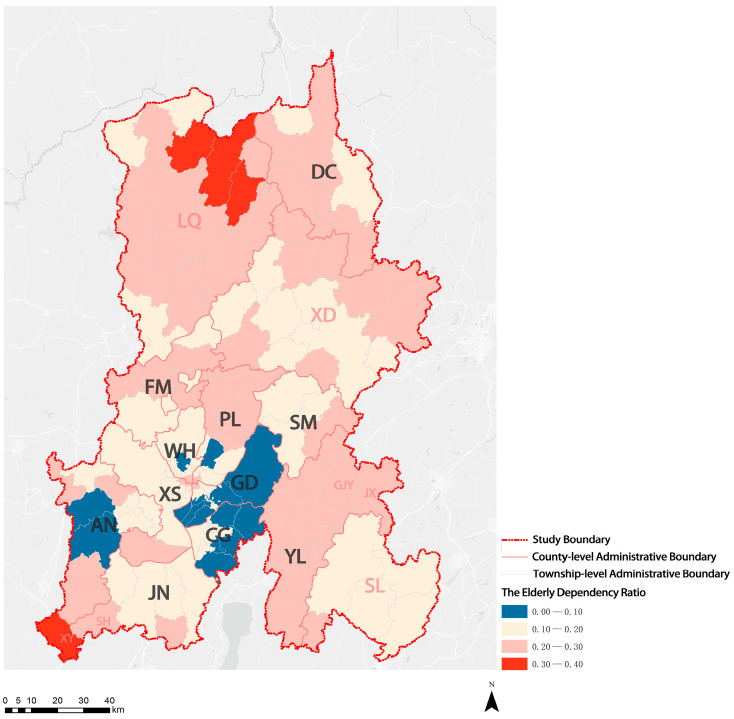
Visualization of distribution results of elderly dependency ratio in KM.

The elderly dependency ratio is an indicator that reflects the social consequences of population aging from an economic perspective. A higher elderly dependency ratio means the working population supports more elderly dependents per capita. In other words, a high elderly dependency ratio signifies a heavier dependency burden on the working population. As shown in Figure 5, the elderly dependency ratio varied between ethnic minority communities and other areas. The mean value of the elderly dependency ratio in all townships (subdistricts) in KM was 0.18. The mean value of the elderly dependency ratio in ethnic minority communities was 0.22, which is higher than that in other areas (0.17). The XY ethnic township and three townships in the LQ Autonomous County had high elderly dependency ratios, with three young persons for every elderly person needed to provide sufficient support.

### 3.2. Service Level of Elderly Care Institutions

In this study, on the basis of open data, the fundamental service level and the convenience degree of elderly care institutions were analyzed, providing a comprehensive evaluation of the service level of elderly care institutions in KM.

#### 3.2.1. Status of Elderly Care Institutions in KM

**Figure 6:**
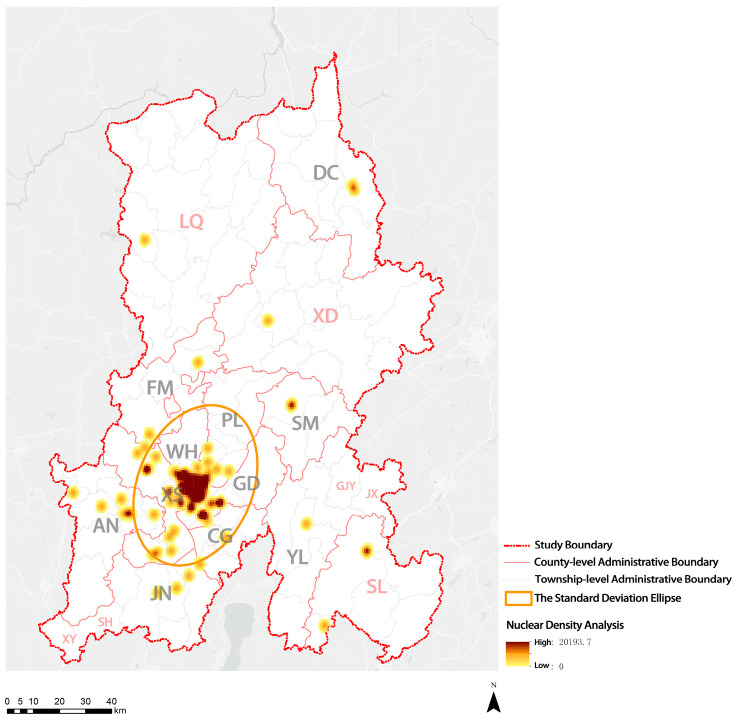
Distribution of elderly care institutions in KM.

This study analyzed the distribution of elderly care institutions in KM. At present, KM has 126 licensed elderly care institutions, including 52 state-run elderly care institutions, 73 privately owned elderly care institutions, and 1 other type of elderly care institution. The results of the standard deviation ellipse (Figure 6) show that most of the elderly care institutions in KM were located within the First Ring Road at the intersection of the four central urban areas (XS District, PL District, GD District, and WH District).

As shown in Table 1, the main types of elderly care institutions in KM were adult homes and independent living apartments, with 86 in total. There were a total of 40 social welfare institutions, nursing homes, sanatoriums, continuing care retirement communities, retirement homes, senior living communities, and other elderly care institutions. In addition, most elderly care institutions only offer self-care services. Elderly care institutions that offered two or more care types were almost all concentrated in central urban areas (XS District, PL District, GD District, and WH District) and the AN County-Level City (Table 2). Almost all elderly care institutions in ethnic minority communities were state-run institutions and suffered from an insufficient quantity and types of care.

#### 3.2.2. Fundamental Service Level of Elderly Care Institutions

The fundamental service level of elderly care institutions was evaluated in this study mainly considering the number of elderly care institutions per 1000 elders, the number of institutional beds per 1000 elders, and the number of workers per 1000 elders. Overall, the number of elderly care institutions per 1000 elders in KM was 0.14, which is much lower than that in Osaka Prefecture in Japan, which has a well-developed elderly care experience (the number of elderly care institutions per 1000 elders is approximately 1.03) [30]. The number of institutional beds per 1000 elders in KM was approximately 35, which is still much lower than that in Osaka Prefecture (54 beds per 1000 elders). Moreover, the number of workers per 1000 elders was fewer than 8, significantly fewer than that in Osaka Prefecture (76 workers per 1000 elders). The results suggest that, on the whole, KM presents an obvious shortage of elderly care institutions, institutional beds, and related workers.

**Figure 7:**
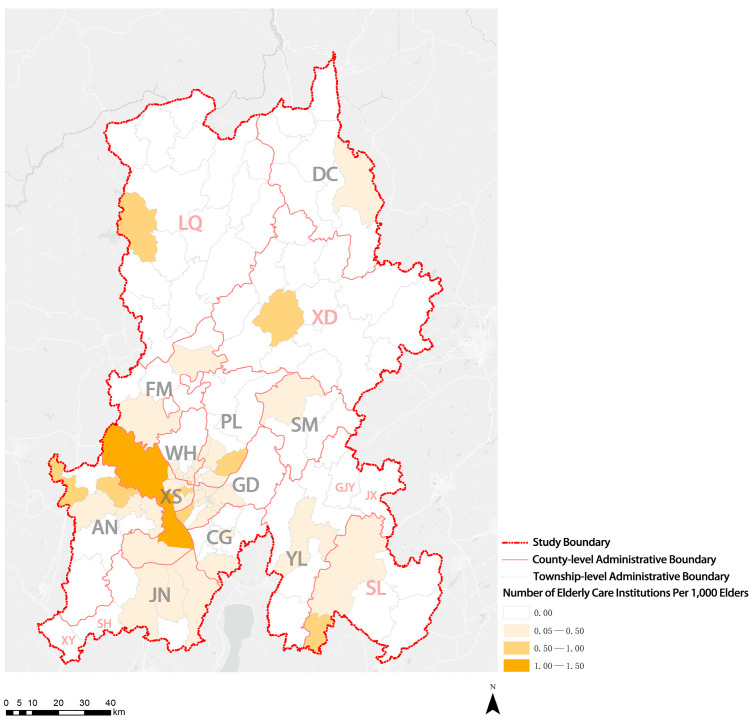
Visualization of distribution results of the number of institutions per 1000 elders.

**Figure 8:**
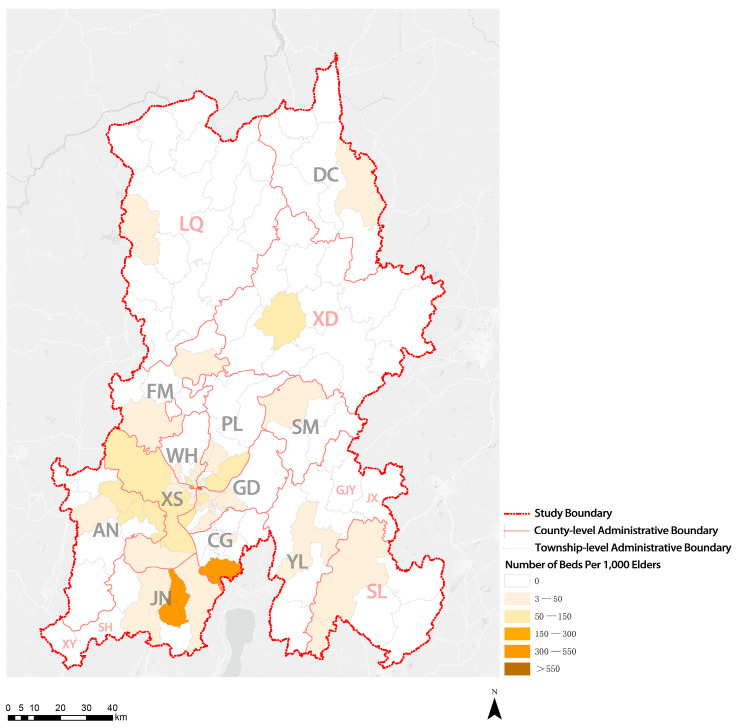
Visualization of distribution results of the number of institutional beds per 1000 elders.

**Figure 9:**
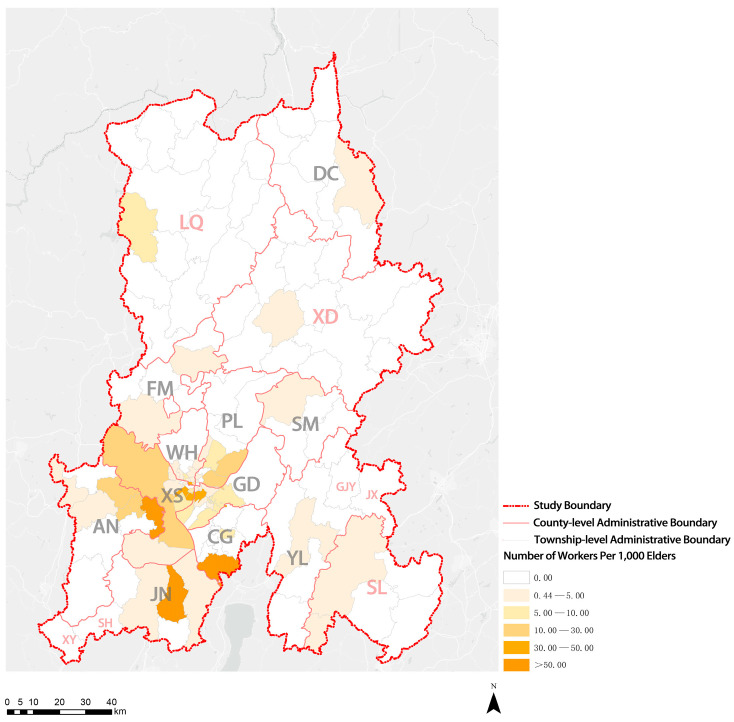
Visualization of distribution results of the number of workers per 1000 elders.

As shown in Figure 7, in KM, only some subdistricts of XS District had more than 1.0 institutions per 1000 elders. Most townships (subdistricts), especially ethnic minority communities, did not have elderly care institutions. Only four streets had more than 300 beds per 1000 elders, and only one street had more than 550 beds per 1000 elders. The number of institutional beds on most streets in the four central urban areas ranged between 50 and 150 beds per 1000 elders, indicating a noticeable shortage of beds for elderly care institutions. The number of institutional beds in areas outside the main urban areas, especially ethnic minority communities, was fewer than 50 beds or was no beds per 1000 elders (Figure 8). Moreover, the analysis results suggest that eight townships (subdistricts) in the whole city had more than 30 workers per 1000 elders, and only four townships (subdistricts) had more than 50 workers per 1000 elders. Given their small local populations and large number of elderly care institutions, two townships (subdistricts) showed more workers per 1000 elders than the other townships (subdistricts). Five to ten workers were assigned per 1000 elders in the four central urban areas, but fewer than five or no workers were assigned in ethnic minority communities (Figure 9). The results suggest that the shortage of elderly care institutions, institutional beds, and workers in ethnic minority communities is more serious.

#### 3.2.3. Comparison between State-Run and Privately Owned Elderly Care Institutions

Privately owned elderly care institutions outnumbered state-run elderly care institutions by approximately 1.4 times (Table 2). State-run elderly care institutions are social welfare institutions subsidized by the government. Therefore, state-run elderly care institutions are evenly distributed in all districts in the whole city. Privately owned elderly care institutions are self-financing enterprises that need substantial capital investment. Thus, most privately owned elderly care institutions were concentrated in the four most economically developed central urban areas (XS District, PL District, GD District, and WH District) to sustain their operation.

Overall, compared with state-run elderly care institutions, privately owned elderly care institutions had better infrastructure. The majority of state-run elderly care institutions were adult homes, and 36.54% were other types of institutions. Among privately owned elderly care institutions, adult homes accounted for 30.14%, independent living apartments accounted for 31.51%, and other types of institutions accounted for 38.35%. Additionally, the average number of beds, nursing prices, and the number of workers in privately owned elderly care institutions were higher than those in state-run elderly care institutions. Moreover, the types of care provided in privately owned elderly care institutions were more diverse. Specifically, the average number of beds in state-run elderly care institutions was about 102, while that in privately owned elderly care institutions was about 350. The average number of beds in privately owned elderly care institutions was about 3.43 times that in state-run elderly care institutions. Additionally, the average price of nursing care in privately owned elderly care institutions was about 3.05 times that in state-run elderly care institutions. Furthermore, the average number of workers in privately owned elderly care institutions (79) was about 4.39 times that in state-run elderly care institutions (18). Care type Ⅰ (Table 2) accounted for 90.38% of care types in state-run elderly care institutions. For privately owned elderly care institutions, 45.20% of institutions offered care type I, 20.55% of institutions offered care type VI, 9.59% of institutions offered care type VII, and 24.66% offered other care types.

#### 3.2.4. Convenience Degree of Elderly Care Institutions

**Figure 10:**
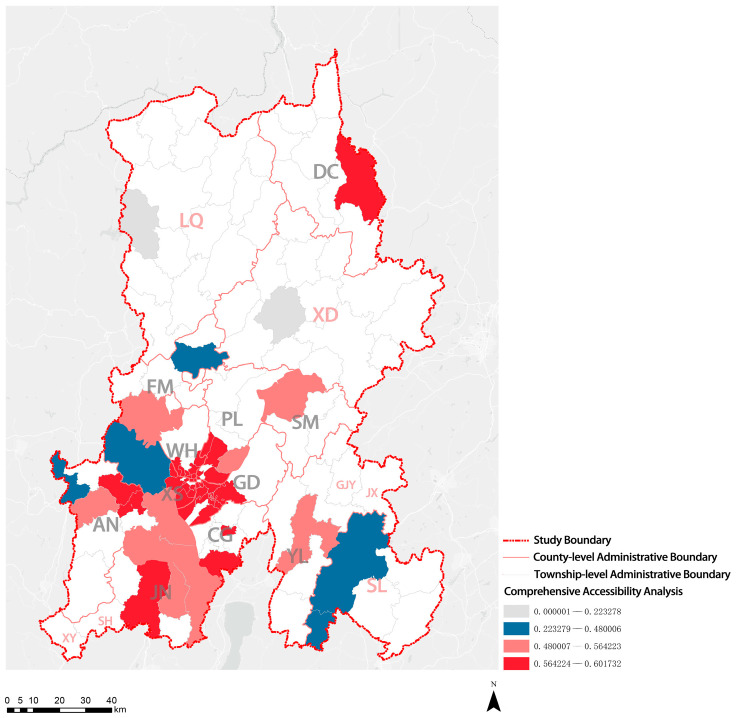
Visualization of distribution results of convenience analysis of elderly care institutions.

POI service facilities include all kinds of engineering and social service facilities in urban spaces, which can be used for various urban studies. The accessibility of POI service facilities was analyzed to evaluate the convenience degree of elderly care institutions in this study. First, the accessibility of each elderly care institution to eight POI service facilities (healthcare, scenic spot, public facility, life service, shopping service, education, science and culture, sports and leisure, and transportation service; Table 3) was calculated. Then, the entropy method was used to assign the index weight to calculate the comprehensive accessibility of each elderly care institution. Finally, the data were visualized. Figure 10 shows the visualized accessibility of POI service facilities in KM. XS District, PL District, GD District, WH District, CG District, JN District, and AN County-Level City had higher comprehensive accessibility than DC District, SM County, YL County, and FM County. XD Autonomous County, SL Autonomous County, and LQ Autonomous County had the lowest comprehensive accessibility. Compared with that in other areas in KM, the convenience degree of elderly care institutions in ethnic minority communities was poor, reflecting an insufficient number and the unreasonable distribution of POI service facilities. Additionally, the comprehensive accessibility of privately owned elderly care institutions to POI service facilities was higher than that of state-run elderly care institutions.

### 3.3. Coupling Coordination Degree

This study analyzed the adaptation of the degree of population aging and the service level of elderly care institutions in KM using the coupling coordination model. On the basis of previous studies, evaluation indicators for the service level of elderly care institutions and population aging were proposed (Table 4). Then, the entropy method was used to calculate the weights of the indicators with respect to the degree of population aging and the service level of institutions. The total score of the degree of population aging and the service level of institutions was obtained according to indicator weights. Finally, the coupling degree C value, coordination index T value, and coupling coordination degree D value, measured using the coupling coordination model, were introduced to characterize the adaptation of the service level of elderly care institutions and the degree of population aging. As shown in Table A2, the coupling coordination degree was classified into 10 levels.

**Figure 11:**
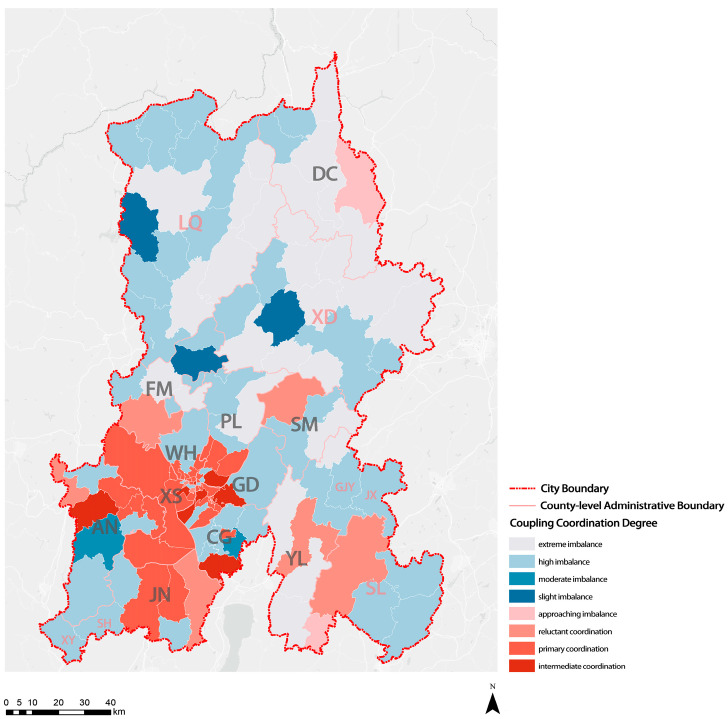
Visualization of distribution results of coupling coordination between the degree of population aging and service level of elderly care institutions.

The calculation results for the coupling coordination between the degree of population aging and the service level of elderly care institutions in KM are shown in Table A3. Fewer than half of the townships (subdistricts) reached reluctant, primary, or intermediate coordination for the degree of population aging and the service level of elderly care (Figure 11). These townships (subdistricts) were mainly under the jurisdiction of seven districts (GD District, WH District, XS District, PL District, JN District, CG District, and DC District). Except for those in DC District, the degree of population aging and the service level of elderly institutions in six districts in KM were comparatively coordinated. The coupling coordination degree of the population aging and the service level of elderly care institutions for the seven districts ranked from high to low followed the order: GD District, WH District, XS District, PL District, JN District, CG District, and DC District. Most townships (subdistricts) in the AN County-Level City showed intermediate coordination or a moderate imbalance in the degree of population aging and the service level of elderly care institutions. Most townships (subdistricts) in SM County and SL Autonomous County showed a moderate imbalance in the degree of population aging and the service level of elderly care institutions. Most townships (subdistricts) in FM County, YL County, LQ Autonomous County, and XD Autonomous County showed a serious mismatch in the area’s current degree of population aging and the coupling coordination degree with a moderate or high imbalance. D values for the above counties were generally low. Twenty-eight townships (subdistricts) showed a high imbalance in the degree of population aging and the service level of elderly care institutions, of which sixteen were under the jurisdiction of XD Autonomous County and LQ Autonomous County. Overall, the imbalance in the coupling coordination between the degree of aging and the service level of elderly care institutions in ethnic minority communities was severe.

## 4. Conclusions

This study took the multi-ethnic city KM as an example to analyze the equity of elderly care facility allocation at a township (subdistrict) scale. The study found the following. ① Regional differences can be seen in the population aging. There was a significant shortage of young people in economically underdeveloped ethnic minority communities, which led to serious population aging. Although the population aging in central urban areas was relatively low, it was constrained by the limited space; thus, the density of elders in central urban areas is considerable. ② The distribution of elderly services in different districts or counties was imbalanced. There were significant differences between urban and rural areas. Elderly service resources were largely centered in the four central urban areas, while all ethnic minority communities were located in counties and townships. Therefore, the number of elderly care institutions in ethnic minority communities, institutional beds, and workers per 1000 elders fell significantly short. ③ The overall convenience of elderly care institutions was low. Relevant service facilities were insufficient and unevenly distributed. A significant difference was seen in the comprehensive accessibility to POI facilities between ethnic minority communities and other areas in KM, indicating a lack of equity. ④ Compared with state-run elderly care institutions, privately owned elderly care institutions offered more types of care, better infrastructure, and better comprehensive accessibility to POI service facilities. ⑤ The coupling coordination between the degree of aging and the service level of elderly care institutions in the majority of the areas in KM showed poor adaptation. Furthermore, the coupling coordination degree in most ethnic minority communities was imbalanced.

Based on the above study results, the following recommendations are proposed:

(1) Elderly care resources should be allocated more equitably. More attention should be paid to ameliorating the unreasonable distribution of public infrastructure. Furthermore, the spatial distribution of the population should be managed via the rational layout of public infrastructure to alleviate excessive aging in the regional population.
(2) The elderly care facilities in ethnic minority communities should be improved. Factors, including the economy, population, ethnic culture, and religious beliefs of ethnic minorities should be considered to construct elderly care institutions with suitable care types. The coverage, number of beds, and number of workers in state-run elderly care institutions should be improved. Considering the unique ethnic culture and ecological environment of ethnic minority communities, elderly care institutions can be developed in such a way to attract urban elders and stimulate the economy in ethnic minority areas, promoting the gradual improvement of elderly care facilities for ethnic minorities.
(3) More public funds and resources should be allocated to ethnic minority communities to improve the equity of allocation. The planning and construction of elderly care institutions should also consider the characteristics of minority nationalities and accommodate the elderly’s care needs in terms of religious beliefs, dietary habits, interior decoration style, and customs to optimize elderly care for the ethnic minorities.

## Figures and Tables

**Table 1:** Types of elderly care institutions in KM.

Administrative District Institution Type	Social Welfare Institution	Nursing Home	Adult Home	Independent Living Apartment	Sanatorium	Continuing Care Retirement Community	Retirement Home	Assisted Living	Others	Total
WH District	1	0	10	6	1	1	1	0	4	24
PL District	0	0	7	3	0	0	1	1	1	13
GD District	0	1	9	12	0	0	0	0	5	27
XS District	0	0	18	9	1	0	4	0	6	38
DC District	1	0	0	0	0	0	1	0	0	2
CG District	0	0	1	0	0	0	0	1	0	2
JN District	0	0	1	0	0	0	1	1	0	3
FM County	0	0	2	0	0	0	0	0	0	2
YL County	0	0	0	0	0	0	1	0	0	1
SM County	0	0	2	0	0	0	0	0	0	2
SL Autonomous County	0	0	2	0	0	0	0	0	1	3
LQ Autonomous County	0	0	1	0	0	0	0	0	0	1
XD Autonomous County	1	0	0	0	0	0	0	0	0	1
AN County-Level City	0	0	2	1	1	0	0	0	3	7
Total	3	1	55	31	3	1	9	3	20	126

**Table 2:** Organization type and care type of the involved elderly care institutions.

Administrative Districts	Organization Type				Care Type							
	State-Run	Privately Owned	Others	Total	I	II	III	IV	V	VI	VII	Total
WH District	11	12	1	24	19	1	1	0	0	2	1	24
PL District	4	9	0	13	7	1	3	0	1	1	0	13
GD District	7	20	0	27	16	0	2	1	1	6	1	27
XS District	14	24	0	38	23	1	5	0	0	5	4	38
DC District	2	0	0	2	1	0	0	0	1	0	0	2
CG District	1	1	0	2	1	0	0	0	0	0	1	2
JN District	1	2	0	3	1	0	0	0	0	1	1	3
FM County	2	0	0	2	2	0	0	0	0	0	0	2
YL County	1	0	0	1	1	0	0	0	0	0	0	1
SM County	2	0	0	2	2	0	0	0	0	0	0	2
SL Autonomous County	2	1	0	3	2	1	0	0	0	0	0	3
LQ Autonomous County	1	0	0	1	1	0	0	0	0	0	0	1
XD Autonomous County	1	0	0	1	1	0	0	0	0	0	0	1
AN County-Level Cities	3	4	0	7	3	0	0	0	0	4	0	7
Total	52	73	1	126	80	4	11	1	3	19	8	126

I self-care; II self-care and half-care; III self-care, half-care, and full care; IV half-care, full care, and special care; V self-care, half-care, and special care; VI self-care, half-care, full care, and special care; VII self-care, half-care, full care, special care, and specialized care.

**Table 3:** Selected POI service facilities around elderly care institutions.

Service	Facility
Healthcare	General hospitals, specialized hospitals, clinics, emergency centers, disease prevention institutions, pharmacies
Scenic spot	Park square, scenic spot
Public facility	Newsstands, public telephones, public toilets, emergency shelters
Life service	Daily services, travel agencies, information centers, ticket offices, post offices, express services, telecommunication offices, offices, water offices, electric power offices, beauty salons, repair stations, photography and printing stores, bath and massage facilities, laundries, funeral facilities
Shopping service	Shopping-related places, shopping malls, convenience stores, home appliance stores, supermarkets, flower stores, pet markets, home building material markets, general markets, cultural goods stores, sporting goods stores, specialty shopping streets, clothing stores, specialty stores, personal goods stores
Education, science, and culture	Science, education and culture places, museums, exhibition halls, convention centers, art galleries, libraries, science and technology museums, planetariums, cultural palaces, archives, literary and artistic groups
Sports and leisure	Sports and leisure services, sports venues, golf-related, entertainment venues, vacation retreats, leisure venues, movie theaters
Transportation service	Subway stations, bus stops, drop-off, pick-up areas

**Table 4:** Evaluation indicators for the service level of elderly care institutions and the degree of population aging.

Tier 1 Indicators	Tier 2 Indicators	Tier 3 Indicators	Weight	Data Source
Service level of senior care institutions	Fundamental service level	Number of elderly care institutions per 1000 elders	0.090	Linkolder and Elderly Care Information Network
		Number of beds per 1000 elders	0.102	
		Number of workers per 1000 elders	0.109	
		Average price of bed and nursing care fees	0.096	
	Convenience of living	Medical service facilities	0.075	AMAP
		Green space service facilities	0.076	
		Public service facilities	0.076	
		Living service facilities	0.075	
		Shopping service facilities	0.075	
		Scientific, educational, and cultural facilities	0.075	
		Transportation service facilities	0.076	
		Sports and leisure facilities	0.075	
Degree of population aging	Proportion of population	Percentage of population aged 0–14	0.094	Data of the Seventh National Population Census
		Percentage of population aged 15–59	0.353	
		Proportion of population aged 60 or older	0.141	
		Elderly-to-child ratio	0.219	
	Dependency ratio	Total dependency ratio	0.063	
		Child dependency ratio	0.048	
		Elderly dependency ratio	0.082	

## Data Availability

Not applicable.

## Appendix A

**Table A1:** Abbreviations of geographical names in manuscript.

No.	Full Name	Abbreviation	Administrative Districts
1	Kunming City	KM City	City
2	Anning City	AN City	County-level City
3	Songming County	SM County	County
4	Yiliang County	YL County	
5	Fumin County	FM County	
6	Panlong District	PL District	District
7	Wuhua District	WH District	
8	Guandu District	GD District	
9	Xishan District	XS District	
10	Dongchuan District	DC District	
11	Chenggong District	CG District	
12	Jinning District	JN District	
13	Shilin Yi Autonomous County	SL Autonomous County	Autonomous County
14	Xundian Hui Autonomous County	XD Autonomous County	
15	Luquan Miao Autonomous County	LQ Autonomous County	
16	Xiyang Yi Ethnic Township	XY Ethnic Township	Ethnic Township
17	Shuanghe Yi Ethnic Township	SH Ethnic Township	
18	Jiuxiang Yi and Hui Ethnic Township	JX Ethnic Township	
19	Gengjiaying Yi and Miao Ethnic Township	GJY Ethnic Township	

**Table A2:** Coupling coordination level classification criteria.

The Range of D Value	Coupling Coordination Level	Meaning
(0.0–0.1)	1	extreme imbalance
[0.1–0.2)	2	high imbalance
[0.2–0.3)	3	moderate imbalance
[0.3–0.4)	4	slight imbalance
[0.4–0.5)	5	approaching imbalance
[0.5–0.6)	6	reluctant coordination
[0.6–0.7)	7	primary coordination
[0.7–0.8)	8	intermediate coordination
[0.8–0.9)	9	high coordination
[0.9–1.0)	10	quality coordination

**Table A3:** Calculation results of coupling coordination degree between the degree of population aging and the service level of elderly care institutions in KM city.

No.	Coupling Degree C Value	Coordination Index T Value	Coupling Coordination Degree D Value	Level	Coupling Coordination Degree
1	0.929	0.267	0.498	5	approaching imbalance
2	0.989	0.195	0.439	5	approaching imbalance
3	0.776	0.312	0.492	5	approaching imbalance
4	0.891	0.333	0.545	6	reluctant coordination
5	0.943	0.524	0.703	8	intermediate coordination
6	0.968	0.489	0.688	7	primary coordination
7	0.887	0.434	0.62	7	primary coordination
8	0.944	0.502	0.688	7	primary coordination
9	0.92	0.624	0.758	8	intermediate coordination
10	0.778	0.431	0.579	6	reluctant coordination
11	0.849	0.638	0.736	8	intermediate coordination
12	0.956	0.765	0.855	9	high coordination
13	0.951	0.613	0.764	8	intermediate coordination
14	0.978	0.584	0.756	8	intermediate coordination
15	0.983	0.665	0.808	9	high coordination
16	0.983	0.638	0.792	8	intermediate coordination
17	0.977	0.592	0.761	8	intermediate coordination
18	0.933	0.455	0.652	7	primary coordination
19	0.99	0.643	0.798	8	intermediate coordination
20	0.912	0.542	0.703	8	intermediate coordination
21	0.97	0.571	0.745	8	intermediate coordination
22	0.997	0.662	0.812	9	high coordination
23	0.975	0.592	0.76	8	intermediate coordination
24	0.969	0.616	0.773	8	intermediate coordination
25	0.967	0.626	0.778	8	intermediate coordination
26	0.987	0.666	0.811	9	high coordination
27	0.975	0.661	0.803	9	high coordination
28	0.955	0.624	0.772	8	intermediate coordination
29	0.92	0.527	0.696	7	primary coordination
30	0.982	0.658	0.804	9	high coordination
31	0.967	0.568	0.741	8	intermediate coordination
32	0.944	0.521	0.701	8	intermediate coordination
33	0.88	0.522	0.678	7	primary coordination
34	0.977	0.591	0.76	8	intermediate coordination
35	0.981	0.595	0.764	8	intermediate coordination
36	0.973	0.66	0.801	9	high coordination
37	0.954	0.616	0.766	8	intermediate coordination
38	0.95	0.527	0.708	8	intermediate coordination
39	0.971	0.588	0.756	8	intermediate coordination
40	0.886	0.458	0.637	7	primary coordination
41	0.983	0.626	0.784	8	intermediate coordination
42	0.94	0.463	0.66	7	primary coordination
43	0.94	0.529	0.705	8	intermediate coordination
44	0.947	0.562	0.729	8	intermediate coordination
45	0.855	0.586	0.708	8	intermediate coordination
46	0.972	0.604	0.766	8	intermediate coordination
47	0.967	0.602	0.763	8	intermediate coordination
48	0.911	0.57	0.721	8	intermediate coordination
49	0.981	0.585	0.757	8	intermediate coordination
50	0.975	0.611	0.771	8	intermediate coordination
51	0.992	0.662	0.81	9	high coordination
52	0.954	0.568	0.736	8	intermediate coordination
53	0.631	0.045	0.168	2	high imbalance
54	0.572	0.056	0.178	2	high imbalance
55	0.554	0.06	0.182	2	high imbalance
56	0.597	0.05	0.174	2	high imbalance
57	0.941	0.015	0.119	2	high imbalance
58	0.266	0.278	0.272	3	moderate imbalance
59	0.263	0.284	0.273	3	moderate imbalance
60	0.287	0.237	0.261	3	moderate imbalance
61	0.321	0.189	0.246	3	moderate imbalance
62	0.228	0.379	0.294	3	moderate imbalance
63	0.303	0.213	0.254	3	moderate imbalance
64	0.403	0.118	0.218	3	moderate imbalance
65	0.333	0.175	0.242	3	moderate imbalance
66	0.313	0.2	0.25	3	moderate imbalance
67	0.24	0.342	0.287	3	moderate imbalance
68	0.199	0.5	0.315	4	slight imbalance
69	0.259	0.293	0.276	3	moderate imbalance
70	0.375	0.137	0.227	3	moderate imbalance
71	0.264	0.281	0.273	3	moderate imbalance
72	0.283	0.245	0.263	3	moderate imbalance
73	0.202	0.484	0.313	4	slight imbalance
74	0.243	0.334	0.285	3	moderate imbalance
75	0.296	0.223	0.257	3	moderate imbalance
76	0.651	0.042	0.164	2	high imbalance
77	0.408	0.115	0.216	3	moderate imbalance
78	0.477	0.082	0.198	2	high imbalance
79	0.506	0.073	0.192	2	high imbalance
80	0.617	0.047	0.17	2	high imbalance
81	0.391	0.125	0.222	3	moderate imbalance
82	0.384	0.13	0.224	3	moderate imbalance
83	0.43	0.103	0.21	3	moderate imbalance
84	0.517	0.07	0.19	2	high imbalance
85	0.436	0.1	0.209	3	moderate imbalance
86	0.474	0.084	0.199	2	high imbalance
87	0.548	0.061	0.183	2	high imbalance
88	0.549	0.061	0.183	2	high imbalance
89	0.649	0.042	0.165	2	high imbalance
90	0.595	0.051	0.174	2	high imbalance
91	0.457	0.091	0.203	3	moderate imbalance
92	0.755	0.029	0.148	2	high imbalance
93	0.471	0.085	0.2	2	high imbalance
94	0.64	0.043	0.166	2	high imbalance
95	0.41	0.114	0.216	3	moderate imbalance
96	1	0.01	0.1	2	high imbalance
97	0.363	0.146	0.231	3	moderate imbalance
98	0.431	0.102	0.21	3	moderate imbalance
99	0.478	0.082	0.198	2	high imbalance
100	0.435	0.1	0.209	3	moderate imbalance
101	0.352	0.156	0.234	3	moderate imbalance
102	0.502	0.074	0.193	2	high imbalance
103	0.34	0.168	0.239	3	moderate imbalance
104	0.344	0.164	0.237	3	moderate imbalance
105	0.314	0.197	0.249	3	moderate imbalance
106	0.359	0.15	0.232	3	moderate imbalance
107	0.313	0.2	0.25	3	moderate imbalance
108	0.556	0.059	0.182	2	high imbalance
109	0.328	0.181	0.244	3	moderate imbalance
110	0.457	0.09	0.203	3	moderate imbalance
111	0.395	0.123	0.22	3	moderate imbalance
112	0.314	0.198	0.249	3	moderate imbalance
113	0.347	0.161	0.236	3	moderate imbalance
114	0.346	0.162	0.237	3	moderate imbalance
115	0.408	0.115	0.217	3	moderate imbalance
116	0.468	0.086	0.201	3	moderate imbalance
117	0.481	0.081	0.198	2	high imbalance
118	0.395	0.123	0.22	3	moderate imbalance
119	0.856	0.021	0.133	2	high imbalance
120	0.461	0.089	0.202	3	moderate imbalance
121	0.397	0.121	0.22	3	moderate imbalance
122	0.409	0.115	0.216	3	moderate imbalance
123	0.476	0.083	0.199	2	high imbalance
124	0.381	0.132	0.225	3	moderate imbalance
125	0.611	0.048	0.171	2	high imbalance
126	0.283	0.245	0.263	3	moderate imbalance
127	0.473	0.084	0.2	2	high imbalance
128	0.399	0.12	0.219	3	moderate imbalance
129	0.482	0.081	0.197	2	high imbalance
130	0.389	0.127	0.222	3	moderate imbalance
131	0.41	0.114	0.216	3	moderate imbalance
132	0.314	0.198	0.249	3	moderate imbalance
